# *Staphylococcus aureus* lineages associated with a free-ranging population of the fruit bat *Pteropus livingstonii* retained over 25 years in captivity

**DOI:** 10.1038/s41598-022-17835-3

**Published:** 2022-08-05

**Authors:** Kay Fountain, Alberto Barbon, Marjorie J. Gibbon, David H. Lloyd, Anette Loeffler, Edward J. Feil

**Affiliations:** 1 Department of Biology and Biochemistry, University of Bath, Claverton Down, Bath, BA2 7AY UK; 2 Department of Clinical Science and Services, Royal Veterinary College, North Mymms, Hatfield, Hertfordshire AL9 7TA UK; 3 North of England Zoological Society (Chester Zoo), Caughall Road, Upton by Chester, Chester, Cheshire CH2 1LH UK

**Keywords:** Evolution, Microbiology, Microbial genetics, Ecological epidemiology

## Abstract

Conservation of endangered species has become increasingly complex, and costly interventions to protect wildlife require a robust scientific evidence base. This includes consideration of the role of the microbiome in preserving animal health. Captivity introduces stressors not encountered in the wild including environmental factors and exposure to exotic species, humans and antimicrobial drugs. These stressors may perturb the microbiomes of wild animals, with negative consequences for their health and welfare and hence the success of the conservation project, and ultimately the risk of release of non-native organisms into native ecosystems. We compared the genomes of *Staphylococcus aureus* colonising critically endangered Livingstone’s fruit bats (*Pteropus livingstonii*) which have been in a captive breeding programme for 25 years, with those from bats in the endemic founder population free ranging in the Comoros Republic. Using whole genome sequencing, we compared 47 isolates from captive bats with 37 isolates from those free ranging in the Comoros Republic. Our findings demonstrate unexpected resilience in the bacteria carried, with the captive bats largely retaining the same two distinctive lineages carried at the time of capture. In addition, we found evidence of genomic changes which suggest specific adaptations to the bat host.

## Introduction

Wildlife conservation projects which aim to protect critically endangered species often involve periods of captivity, leading to stresses not encountered in the wild^1^. A large body of work has highlighted the importance of the microbiome to human and animal health, including efficient immune function, dietary provision and protection against pathogens and it is now recognised that the suite of colonising microorganisms should likewise be conserved^2–4^. Captive animals experience a change in diet and environment, as well as exposure to exotic species (including humans) and antimicrobials, any of which may cause indirect harm through perturbations to the animal’s resident microflora and threaten the chances of successful release^5^. However, little is known about how these microbial communities change as a result of captivity, nor what the consequences of this may be^6^.

Bats belong to the order *Chiroptera*, which includes more species than any other mammalian order, but also has the greatest number of species at risk^7^. Livingstone’s fruit bats (*Pteropus livingstonii*) are the second largest bat species in the world, and are critically endangered; the wild population is restricted to the Comoros Republic and estimated to consist of only 1500 individuals^7,8^. Between 1993 and 1995, as part of a species recovery plan, 17 bats were captured on the island of Anjouan to establish a captive breeding population in Jersey Zoo (Durrell Wildlife Conservation Park, U.K.)^9^. Descendants of this population were transferred to, and occasionally back from, Bristol Zoological Gardens (UK), Zoo Zurich (Switzerland), Paris Zoo (France) and Chester Zoo (UK). At the time of this study (2015–2019), this captive colony consisted of around 45 bats, made up of two of the original wild caught bats and 43 descendants of the founding colony, the maternal pedigrees of which are known. This separation, of approximately 25 years, of the captive colony and the free-ranging population from which it was drawn, provides a unique opportunity to investigate the resilience of their native microflora, and the extent to which skin infections and lesions in the captive bats results from the acquisition of *Staphylococcus aureus* isolates not encountered in the wild.

The Firmicute species *Staphylococcus aureus* is a key member of the bacterial microflora of the skin, oral cavity and gut of both humans and a wide range of wild, domesticated and captive wild animals^10–15^. There is convincing evidence of multifarious host adaptations in this species, involving large-scale changes in gene content, or more subtle changes in coding sequences^16,17^. *S. aureus* is also an opportunistic pathogen, and is an important cause of skin and soft-tissue infections, osteomyelitis, endocarditis and septicaemia in both humans and animals^10,18^, including within the bat colony at Jersey zoo^19^. *S. aureus* therefore provides an excellent model to examine the extent to which the resident, and potentially adaptive, microflora in captive animals might be perturbed due to antibiotic therapy, or else forced to compete against potentially pathogenic strains carried by co-residing species or their human contacts.

Here we use whole genome sequencing to compare *S. aureus* isolates from captive Livingstone’s fruit bats in Jersey Zoo, with those from the free-ranging population in the Comoros Republic from which this captive colony was founded 25 years previously. The same two distinct *S. aureus* lineages were found circulating both in the free-ranging and captive populations. This demonstrates that animal microbiomes can be vertically inherited and resiliently maintained over many years of captivity. One explanation would be that these two lineages possess a competitive advantage over any invading strains, a view supported by convergent genomic changes related to immune evasion and carbohydrate metabolism that point to specific adaptations to the bat host.

## Results and discussion

We assembled and sequenced a collection of 84 *S. aureus* isolates recovered between 2015 and 2019 from Livingstone’s fruit bats (Table 1). They included 47 from the captive bat colony on Jersey and 37 collected from the wild roost site in Mohéli (Comoros Republic) (Table S1 for full details including identifiers for individual bat sources). All 45 captive bats were sampled, some from multiple body sites. Isolates were also recovered from their enclosure and from skin lesions. To avoid unacceptable handling and stress to endangered animals, *S. aureus* from the free-ranging bats were obtained using a non-invasive method, first validated in the captive bat colony, whereby *S. aureus* isolates were recovered from faeces and mouth ejecta (chewed and discarded fruit) (“Methods and materials”).

**Table 1:** *Staphylococcus aureus* genomes sequenced from captive Livingstone’s fruit bats in Jersey Zoo and free-ranging in Mohéli, Comoros Republic.

Bat host information			Number of isolates of each ST			
Population type	Location	Sampling site	ST1	ST3926	ST1460	Total
Captive	Jersey Zoo	Skin	4	5	1	10
		Oropharynx	2	3	0	5
		Mouth ejecta	6	2	0	8
		Faeces	2	1	0	3
		Environment	1	0	0	1
		Lesions	15	4	1	20
Free-ranging	Comoros	Faeces	20	14	0	34
		Mouth ejecta	1	2	0	3
					Total sequences	84

Number of sequence types (ST) acquired from each population and sample site.

The *S. aureus* genome sequences revealed a low level of diversity in both bat populations. Two sequence types, ST1 and ST3926 (a novel single locus variant of ST188), accounted for 82/84 (97.6%) of all isolates. The exceptions were two isolates from a single captive bat (a founding member of the colony) that corresponded to the singleton ST1460. The frequencies of the two dominant STs were not significantly different in the captive and free-ranging populations, and there is no clear evidence that the STs differ with respect to source (Table 1), or that either of these lineages are disproportionately associated with disease.

In order to place ST1 and the ST3926 isolates into a phylogenetic context, we assembled a collection of genome sequences from public databases corresponding to close relatives of these STs belonging to the same clonal complex (CC). We identified 316 close relatives of ST1 (CC1) and 145 close relatives of ST3926 (CC188) (this latter collection included five ST188 isolates from red squirrels^20^).

**Figure 1:**
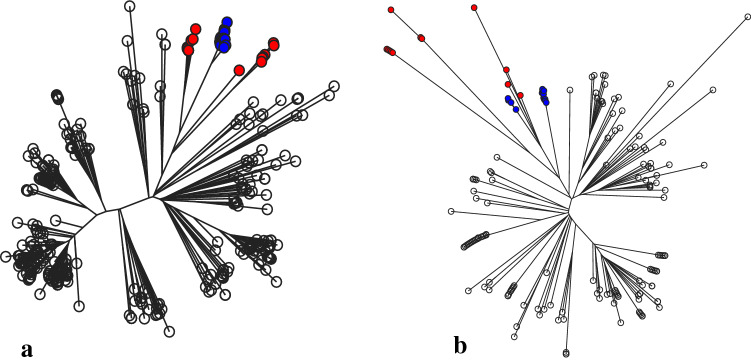
Phylogenies based on an alignment of *S. aureus* core genes from CC1 isolates (**a**) and CC188 isolates (**b**). Isolates from bats in captivity are shown in blue, isolates from free-ranging bats are shown in red. Other branches correspond to lineages from public databases. These trees are available to explore on Microreact: (**a**) https://microreact.org/project/fz62ZcddcfJmv7AaAhTGut-s-aureus-st1-in-livingstones-bats. (**b**) https://microreact.org/project/qSKkpazZp7qpeGWtSY9ocg-s-aureus-cc188-in-livingstones-bats.

Phylogenetic analyses based on core genome SNPs (“Methods and materials”) revealed that in both clonal complexes the isolates from captive and free-ranging bats are closely related and form distinct sub-clusters within the broader tree (Fig. 1a; CC1 and Fig. 1b; CC188). As these two bat populations have had no contact since the captive colony was founded, the most plausible explanation for this is that the isolates circulating in the captive population at the time of sampling are descended from those present in the original wild-caught bats that founded the colony. Thus, despite multiple stresses over 25 years, the *S. aureus* isolates have both been retained by the founding bats, and inherited by the bats subsequently born into the captive colony. It is not possible to compare the prevalence at which these lineages are carried in the captive versus the free-ranging population, as we do not have data for individual free-ranging bats, and it is therefore possible that the carriage rate has decreased in the captive population. Nevertheless, 15/45 of the bats (33%) carried these S. aureus lineages, a carriage rate which is similar to other species, including humans. We note that ST1 isolates from the captive bats correspond to a single monophyletic cluster arising from within the isolates from the free-ranging population (Fig. 1a). In contrast, two distinct clusters are noted within the ST3926 isolates from captive bats (Fig. 1b). Whilst it is possible that these two clusters have diversified subsequent to the founding of the captive colony, the increased average pairwise SNP distance between the ST3926 isolates from captive bats, compared to the ST1 isolates (see below), suggest that these two clusters had already diversified in the free-ranging population and were incidentally sampled in the original captured bats.

For both lineages, isolates from the captive population were less diverse than those from the free-ranging population. For ST1, average pairwise SNP distances were 37.7 SNPs within the captive population compared to 514 SNPs amongst isolates from the free-ranging colony; for ST3926, SNP distances averaged 166.4 SNPs in the captive and 750.6 SNPs in the free-ranging populations. Whilst this is consistent with a founder effect owing to limited diversity amongst the 17 original wild-caught bats, it may also partly reflect transmission within this small colony, combined with the fact that multiple isolates from the same individual captive bats were sequenced.

**Figure 2:**
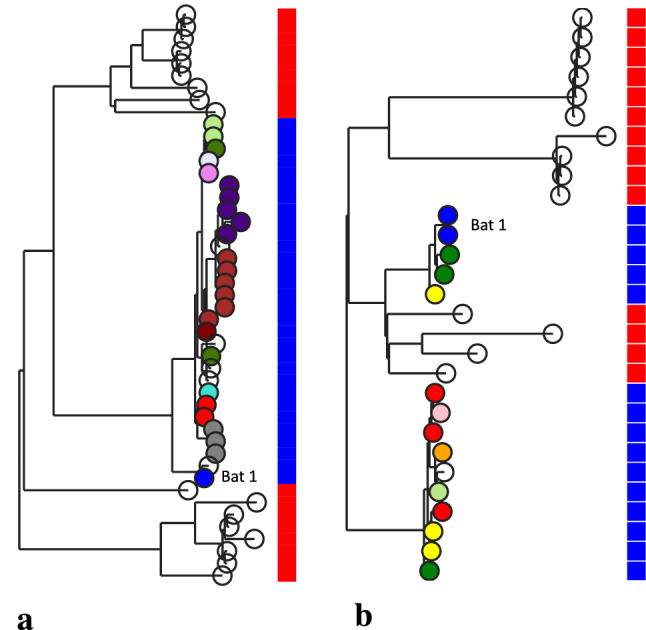
Phylogeny of isolates recovered from captive (coloured nodes, blue squares) and free-ranging bats (open nodes, red squares). For the former, colours of the nodes represent the individual captive bats. ST1 isolates are shown in (**a**), and ST3926 isolates are in (**b**). Clusters of very similar ST1 isolates are noted in the ‘purple’ and ‘brown’ bats. In some cases individual bats harboured isolates corresponding to different clusters. Isolates from one of the original founding bats are indicated (Bat 1).

To explore the possibility of transmission within the captive colony, we considered the phylogenies for the captive bat isolates only (Fig. 2a,b). This revealed the presence of very closely related isolates within different individuals, consistent with transmission within the colony. Bat 1 (one of the original captured bats) harbours a slightly divergent ST 1 isolate, but a typical ST3926 isolate, suggesting this latter isolate has been acquired from other bats in the captive colony. There are also contrasting cases where isolates from the same individual bat correspond to clusters on the tree (eg the ‘purple’ and ‘brown’ individuals from the ST1 tree), consistent with individuals being colonised by a single dominant sub-lineage. These observations align with the low average pairwise diversity in these isolates compared to those from the free-ranging population.

Whilst the phylogenetic evidence points to inheritance and retention of the two dominant lineages ST1 and ST3926 from the wild, the provenance of ST1460, which was not detected in the free-ranging population, is less clear. The two isolates corresponding to this ST were recovered from a carriage and an infection site of the same captive, original founder bat (Bat 1). Inspection of the genome sequence of the ST1460 isolates revealed a beta-haemolysin splitting phage carrying the immune evasion complex genes *sak*, *sell*, *scn*, *sea* and *sec*. These genes were not detected in any other isolates from either bat population, and are characteristic markers of human associated *S. aureus*^21,22^. This therefore raises the possibility that the ST1460 isolates are not typically bat-associated but may have been acquired from a human.

The phylogenetic analysis described above is based on core genome variation. We also considered differences in the accessory genome between the captive and free-ranging populations, and in particular the plasmid content using Mob-Suite (“Methods and materials” and Table S2). Fourteen plasmids were detected in all isolates, of which 11 are novel. These plasmids are highly non-randomly distributed between the free-ranging and captive populations. Only 3/47 isolates (6.4%) from captive bats are predicted to contain any plasmids, compared to 35/37 (94.6%) in the free-ranging population. There was some overlap in the plasmid content of the two lineages based on the short-read data, for example replicon clusters 1017 and 1230 (Table S2). We then considered the genomes constructed from long-read data (one from each lineage) and found one plasmid that is identical in both lineages, plus two further complete plasmids which mirrored the short-read data. None of the plasmids carried antimicrobial resistance genes, but several carried genes predicted to produce serine-aspartate repeat containing proteins (*sdrC*, *sdrE*), and the serine protease *splE.* Although these proteins have been shown to be involved in host interaction, their loss in carriage isolates from the captive population implies that they were not essential for maintenance of carriage in the fruit bats^23,24^. It is not clear what the functions of these plasmids are, nor why they have been lost in the isolates from the captive population although changes in environmental conditions and the strong bottleneck experienced by the captive colony may be contributing factors^25^.

*S. aureus* is known to adapt to different animal hosts, and bat-specific adaptations would help to explain the long-term retention of the two distinct lineages^15^. In order to identify genes that are associated with the ST1 and ST 3926 isolates recovered from the bats (which may potentially play a role in the adaptation of these lineages to the bat host), we used a genome-wide association study (GWAS) approach (“Methods and materials”). We excluded the two ST1460 isolates as these are potentially of human origin. The availability of two distinct bat-associated *S. aureus* lineages, corresponding to CC1 and CC188, adds considerable power to the analysis as it provides the means to detect convergent signals, thus at least partially controlling for population structure.

In order to identify unitigs enriched in the bat-associated isolates compared to related isolates from other hosts we performed a phylogeny-independent GWAS analysis separately on all CC1 and CC188 isolates (“Methods and materials”). We then identified the genes corresponding to significant unitig hits and, by comparing the results from the two lineages, found the overlapping set of genes representing hits in both lineages (note that the specific unitigs can be different, even if the genes are the same). This approach identified the 10 genes listed in Table 2. These include two adhesins, *sraP* (which encodes the serine-rich adhesin for platelets) and *ebh* (which encodes an extracellular matrix binding protein) as well as the *plc* gene which enhances the invasion and persistence of *S. aureus* in keratinocytes^26–29^. The other genes are involved in metabolism, including three genes (*lacA, malG*/*malP* and *glpF*/*glpK)* that are involved in carbohydrate metabolism. These genes are particularly noteworthy as carbohydrate metabolism in fruit bats is physiologically different from other mammals and involves wide fluctuations in blood glucose similar to that described in hummingbirds (Family: *Trochilidae*)^30^. Further investigation into the ability of these bat-adapted strains to thrive in a high sugar environment is therefore warranted. We also note that the iron metabolism gene *isdB*, and the virulence factor lipase 2 also correspond to significant hits in both lineages.

**Table 2:** Genes or regions corresponding to unitigs present in all *S. aureus* isolates from bats and in no other host isolates in both ST1 and ST3926 (CC188) with the function (where known).

Gene or region	Function
*sraP* gene or intergenic *sraP* and start of *secY2*	Adhesion
*plc* gene	Interaction with PMN’s in infection
*Ebh*	Adhesion
*menG* gene or intergenic *menB* and *sspC*	Iron metabolism, functions unclear
Intergenic/start of *lacA* gene	Carbohydrate metabolism
*malG* or *malP*	Carbohydrate metabolism
*glpE* gene or intergenic between *glpF* and *glpK*	Carbohydrate metabolism
*yhdN_1* or intergenic *ywqN* and *yhdN_1*	Oxidoreductase metabolism
*korA* gene or *miaB* gene near to *kor* operon	Metabolism oxidoreductase or methylthiotransferase
*codY* gene or *ilvA* gene regulated by *codY* and *ccpA*	Virulence gene regulation or metabolism of amino acids and fatty acids

Although few genes conferring resistance to antibiotics were found, three captive bats in the hospital enclosure carried ST3926 isolates which have acquired the *blaZ* operon and are phenotypically resistant to amoxicillin. This operon is usually found on a mobile genetic element associated with transposon TN552, although this element was not detected in these isolates and these genes may be chromosomal^31^. Clavulanic acid potentiated amoxicillin is the most frequently used antibiotic in the captive bats so there may have been selection pressure to acquire these genes. In addition, the repressor gene *blaI* from the operon has been found to have a role in immune evasion^32^.

## Conclusion

This retrospective study compares *S. aureus* carriage in a captive population of Livingstone’s bats with the flora of the free-ranging population from which it had been drawn 25 years previously. The results demonstrate that the captive bats have retained two naturally colonising lineages of *S. aureus*, and while a single bat acquired an additional strain, most likely from a human, more evidence would be needed to establish whether such cross-species infection is more likely to cause harm. One of the objectives of bio-security in zoos is to protect the exotic species in their care from infection by micro-organisms which are not normally part of their resident microflora; in the case of *S. aureus* this has clearly been achieved for this captive colony of critically endangered bats. Although previous work has shown the retention of strains in rats of wild origin kept in captivity, to our knowledge this is the first time such long-term retention of the natural microbiota has been demonstrated in any captive population, and provides an insight into host adaptation in *S. aureus*^33^. The phylogenies reveal that the captive bats carried these strains at the time of capture and that they continued to circulate, and be vertically inherited, in the colony throughout the years in captivity.

## Methods and materials

### Sample collection

Over the last 25 years, the colony of Livingstone’s bats has been housed in up to five different enclosures together with a colony of Rodrigues fruit bats (*P. rodricencis*). Standard veterinary management included the administration of antibiotics, antiparasiticides, anaesthetics and surgical procedures including dentistry and wound or fracture management. Captive bats in Jersey Zoo were sampled opportunistically between 2015 and 2019 from multiple sites. The ventral wing skin of conscious bats was sampled while they were distracted by a food treat, by rolling a cotton-tipped swab pre-moistened with tryptone soy broth (TSB) (Oxoid, Thermo-Fisher Scientific Ltd, Basingstoke Hampshire, UK) firmly across a 2 cm patch of skin for up to 6 s. The swab was immediately placed into TSB plus 10% salt (Sigma-Aldrich, Gillingham, Dorset, UK) (TSB+) and incubated at 37 °C for up to 48 h. The oropharynx of bats under anaesthesia for other purposes was sampled using a dry cotton-tipped swab. Mouth-ejecta and faeces were sampled by opening freshly voided pellets aseptically and sampling the centre using a dry cotton-tipped swab, and the enclosure furniture such as ropes, food cups and artificial turf flooring was sampled by rubbing a TSB+ moistened swab over an area approximately 3 cm^2^ just before daily enclosure cleaning. Skin lesions were sampled in conscious or anaesthetised bats using dry cotton-tipped swabs.

A 0.1 µl aliquot of incubated TSB+ was streaked onto 5% Columbia sheep blood agar (CBA) (Oxoid) and incubated for 24–48 h. Up to five haemolytic colonies were picked from the plate and subcultured on CBA for a further 24 h before being stored frozen at − 20 °C and − 80 °C. Presumptive *S. aureus* isolates were identified based on haemolysis on CBA and production of DNase on DNase agar (Oxoid).

Free-ranging bats on the island of Moheli in the Republic of Comoros were sampled during a single trip in 2019. Livingstone’s bats roost in mostly (but not exclusively) single species roosts at high altitude where they avoid disturbance from human activity. They nevertheless are exposed to microbes carried by other species such as birds and to contaminated fomites in the arboreal environment. Faeces, mouth ejecta and items of chewed fruit were collected from beneath a single roost in the Livingstone’s bat reserve on two occasions and transferred within 3 h as above to TSB+ for incubation at 37 °C for up to 48 h in a portable incubator. One milliliter of incubated TSB+ was transferred aseptically to a cryovial containing 0.4 ml of 50% sterile glycerol solution (Thermo Fisher Scientific, Paisley, UK) and stored at room temperature for up to fourteen days for return to the UK before streaking out onto CBA as above.

Presumptive *S. aureus* isolates were screened for methicillin resistance using oxacillin-resistance screening agar (ORSAB, Oxoid) and those from captive bats were further tested for antimicrobial resistance (AMR) by disc diffusion on Muller-Hinton agar as described in Fountain et al.^19^.

### Genome sequencing and bioinformatics

Presumptive *S. aureus* isolates were subcultured and sent on beads (Microbank, Prolab Diagnostics, Cheshire, UK) to Microbes NG (Birmingham) for sequencing on the Illumina Hiseq platform using a 250 bp paired end protocol (Illumina, San Diego, USA). The methods used are available at: https://microbesng.com/documents/5/MicrobesNG_Methods_Document_-_PDF.pdf.

Closely related publicly available genomes for each lineage were identified using PubMLST and downloaded from Genbank for comparison^34,35^.

Based on the Illumina data, two isolates were selected for additional long-read sequencing using the Oxford Nanopore platform (Oxford Nanopore Technologies, Oxford, UK). Genomic DNA was extracted using the Wizard DNA Extraction Kit (Promega, UK). Libraries were prepared using the Rapid Barcoding Kit and multiplexed samples were sequenced using a R9.4.1 flow cell on a MinION (Oxford Nanopore Technologies). Reads were demultiplexed using Deepbinner (v0.2.0) followed by hybrid assembly to produce a closed genome using Unicycler (v0.4.8)^36,37^.

Raw Illumina reads were trimmed using Trimmomatic (v0.33) and quality was confirmed with Fastqc (v0.11.8)^38,39^. Reads were assembled using SPAdes (v3.14.0) and annotated with Prokka (v1.14.5)^40,41^. Multi-locus sequence types (MLST) were identified using ‘MLST’ (v2.15.2)^35,42^. Pangenomes were built using Roary (v3.12.0) and phylogenetic trees constructed from the core gene alignments using a maximum-likelihood method as implemented in RAxML-NG (v0.9.0), a discrete GAMMA model of rate heterogeneity with four categories, and 100 bootstrap replicates^43,44^. To refine the clades a mapping alignment was created using Snippy-multi (v4.1.0) and the hybrid assembly of each sequence type as the reference^45^. The resulting whole genome alignment was used to construct phylogenies (Fig. 1a,b). Reads from the poor quality assemblies which had been excluded from the pangenome analysis were included in the mapping.

Genomes were visually examined and manipulated using Artemis (v18.0.2); further alignments were constructed using Clustal Omega (v1.2.4) and viewed using Jalview (v2.10.5)^46–48^.

Abricate (v1.0.0) was used to search for antimicrobial resistance and virulence genes, and plasmid replicons using the incorporated databases ARG-ANNOT, CARD, Resfinder, VFDB and PlasmidFinder^49–54^. A custom database was created from the Phaster website database to search for intact phages (downloaded Aug 2018)^55^. Potential plasmid sequences were confirmed using plasmidSPAdes and investigated using Mob-recon from Mob-suite (v1.4.9) to classify them against the database of known plasmids^56,57^.

A genome-wide association study (GWAS) was run on assemblies using DBGWAS (v0.5.4) on unitigs^58^. As *S. aureus* is highly clonal DBGWAS was run separately on individual lineages without using a phylogeny, then the top hits in each lineage were searched for genes or regions that were common between lineages.

This study was approved by the Royal Veterinary College Clinical Research Ethical Review Board (CRERB 2015 1332), by the Durrell Wildlife Conservation Trust Ethics Committee, and by the University of Bath Animal Welfare & Ethical Review Body. All methods were performed in accordance with relevant guidelines and regulations for experimental subjects and are reported in accordance with ARRIVE guidelines.

## Supplementary Information

Supplementary Tables.

## Data Availability

Data repositories: Genbank Bioproject PRJNA686248 (https://www.ncbi.nlm.nih.gov/bioproject/PRJNA686248/), Closed genome accessions: CP066492-CP066495, CP066488–CP066491 Individual Genbank accession numbers for each genome are listed in Supplementary Table S1.
