# Supplementary material for: Staphylococcus aureus lineages associated with a free-ranging population of the fruit bat Pteropus livingstonii retained over 25 years in captivity
Source: Sci Rep. 2022 Aug 5;12:13457. doi: 10.1038/s41598-022-17835-3 (PMC9355961; doi:10.1038/s41598-022-17835-3)
Supplement: Supplementary file 1 — Supplementary Tables. [file 41598_2022_17835_MOESM1_ESM.pdf]

***Staphylococcus aureus* lineages associated with a free-ranging population of the fruit bat *Pteropus livingstonii* retained over 25 years in captivity. Kay Fountain, Alberto Barbon, Marjorie J Gibbon, David H. Lloyd, Anette Loeffler, Edward J Feil.**

Supplementary Table S1; List of isolates, sampling site, date and location, host type with ID where available, sequence type and Genbank accession number.

Isolate ID	Host	Bat ID	Sampling site	ampling Dat	Location	MLST	Accession Number
18/1-F BHC	Livingstone’s bat	2	Mouth ejecta	18/01/16	Jersey Zoo	3926	JAEMBE000000000
21/11-F1 LY	Livingstone’s bat		Faeces	21/11/17	Jersey Zoo	3926	JAEMBD000000000
22/11-11 HW	Livingstone’s bat	5	Mouth ejecta	22/11/17	Jersey Zoo	3926	JAEMBC000000000
23/2-14 LBH	Livingstone’s bat	3	Skin	23/02/15	Jersey Zoo	3926	JAEMBB000000000
23/2-5 SG	Livingstone’s bat	2	Skin	23/02/15	Jersey Zoo	3926	JAEMBA000000000
23/2-5 YBH	Livingstone’s bat	2	Skin	23/02/15	Jersey Zoo	3926	JAEMAZ000000000
23/2-9 YBH	Livingstone’s bat	4	Skin	23/02/15	Jersey Zoo	3926	JAEMAY000000000
23/2-9 S	Livingstone’s bat	4	Skin	23/02/15	Jersey Zoo	3926	JAEMAX000000000
27/4-1 BHC	Livingstone’s bat	6	Oropharynx	27/04/16	Jersey Zoo	3926	JAEMAW000000000
27/4-11 BHC	Livingstone’s bat	7	Oropharynx	27/04/16	Jersey Zoo	3926	JAEMAV000000000
27/4-24 BHC	Livingstone’s bat	3	Lesion	27/04/16	Jersey Zoo	3926	JAEMAU000000000
27/4-24 LHY	Livingstone’s bat	3	Lesion	27/04/16	Jersey Zoo	3926	JAEMAT000000000
27/4-5 BHC	Livingstone’s bat	4	Oropharynx	27/04/16	Jersey Zoo	3926	JAEMAS000000000
NC LW	Livingstone’s bat	1	Lesion	14/07/15	Jersey Zoo	3926	JAEMAR000000000
NC LWH	Livingstone’s bat	1	Lesion	14/07/15	Jersey Zoo	3926	JAEMAQ000000000
27187_155F132	Livingstone’s bat		Faeces	15/05/19	Comoros	3926	JAEMAP000000000
27180_155F71	Livingstone’s bat		Faeces	15/05/19	Comoros	3926	JAEMAO000000000
27182_155F75	Livingstone’s bat		Faeces	15/05/19	Comoros	3926	JAEMAN000000000
27189_155F174	Livingstone’s bat		Faeces	15/05/19	Comoros	3926	JAEMAM000000000
27178_155F43	Livingstone’s bat		Faeces	15/05/19	Comoros	3926	JAEMAL000000000
27179_155F52	Livingstone’s bat		Faeces	15/05/19	Comoros	3926	JAEMAK000000000
27207_175TM671	Livingstone’s bat		Chewed fruit	17/05/19	Comoros	3926	JAEMAJ000000000
27203_175F433	Livingstone’s bat		Faeces	17/05/19	Comoros	3926	JAEMAI000000000
27202_175F371	Livingstone’s bat		Faeces	17/05/19	Comoros	3926	See closed genome
27208_175TM674	Livingstone’s bat		Chewed fruit	17/05/19	Comoros	3926	JAEMAH000000000
27190_155F201	Livingstone’s bat		Faeces	15/05/19	Comoros	3926	JAEMAG000000000

30411_155F73	Livingstone’s bat		Faeces	15/05/19	Comoros	3926	JAEMAF000000000
30404_155F173	Livingstone’s bat		Faeces	15/05/19	Comoros	3926	JAEMAE000000000
30407_155F163	Livingstone’s bat		Faeces	15/05/19	Comoros	3926	JAEMAD000000000
30409_155F184	Livingstone’s bat		Faeces	15/05/19	Comoros	3926	JAEMAC000000000
30408_155F165	Livingstone’s bat		Faeces	15/05/19	Comoros	3926	JAEMAB000000000
18/1-12 BHC	Livingstone’s bat	8	Lesion	18/01/16	Jersey Zoo	1	JAEMAA000000000
18/1-19 BHC	Livingstone’s bat	9	Skin	18/01/16	Jersey Zoo	1	JAELZZ000000000
18/1-2 BHY	Livingstone’s bat	10	Skin	18/01/16	Jersey Zoo	1	JAELZY000000000
18/1-3 BHC	Livingstone’s bat	7	Skin	18/01/16	Jersey Zoo	1	JAELZX000000000
18/1-3 SG	Livingstone’s bat	7	Skin	18/01/16	Jersey Zoo	1	JAELZW000000000
18/1-D BHC	Livingstone’s bat	11	Mouth ejecta	18/01/16	Jersey Zoo	1	JAELZV000000000
18/1-E BHC	Livingstone’s bat	12	Mouth ejecta	18/01/16	Jersey Zoo	1	JAELZU000000000
21/11-2 LC	Livingstone’s bat	11	Mouth ejecta	21/11/17	Jersey Zoo	1	JAELZT000000000
21/11-5 LC	Livingstone’s bat	1	Mouth ejecta	21/11/17	Jersey Zoo	1	JAELZS000000000
21/11-F5 LY	Livingstone’s bat		Faeces	21/11/17	Jersey Zoo	1	JAELZR000000000
21/11-F8H LC	Livingstone’s bat		Faeces	21/11/17	Jersey Zoo	1	JAELZQ000000000
23/11-13 HW	Livingstone’s bat		Mouth ejecta	23/11/17	Jersey Zoo	1	JAELZP000000000
23/11-14 HW	Livingstone’s bat		Mouth ejecta	23/11/17	Jersey Zoo	1	JAELZO000000000
23/2-17 Whaem	Livingstone’s bat		Skin	23/02/15	Jersey Zoo	1	JAELZN000000000
27/4-18 BHY	Livingstone’s bat	13	Lesion	27/04/16	Jersey Zoo	1	JAELZM000000000
27/4-18 LC	Livingstone’s bat	13	Lesion	27/04/16	Jersey Zoo	1	JAELZL000000000
27/4-18 SC	Livingstone’s bat	13	Lesion	27/04/16	Jersey Zoo	1	JAELZK000000000
27/4-20 AHC	Livingstone’s bat	14	Oropharynx	27/04/16	Jersey Zoo	1	JAELZJ000000000
27/4-24 LC	Livingstone’s bat	3	Lesion	27/04/16	Jersey Zoo	1	JAELZI000000000
27/4-8 LHC	Livingstone’s bat	3	Oropharynx	27/04/16	Jersey Zoo	1	JAELZH000000000
23535_5_3_M3012_3	Livingstone’s bat	10	Lesion	05/03/19	Jersey Zoo	1	JAELZE000000000
23536_5_3_M3012_4	Livingstone’s bat	10	Lesion	05/03/19	Jersey Zoo	1	JAELZD000000000
23537_5_3_M3012_1	Livingstone’s bat	10	Lesion	05/03/19	Jersey Zoo	1	JAELZG000000000
23538_5_3_M3012_2	Livingstone’s bat	10	Lesion	05/03/19	Jersey Zoo	1	JAELZF000000000
23591_5_3_M3012_5	Livingstone’s bat	10	Lesion	05/03/19	Jersey Zoo	1	JAELZC000000000
27173_54M3289Y1	Livingstone’s bat	15	Lesion	05/04/19	Jersey Zoo	1	JAELZB000000000
27174_54M3289Y2	Livingstone’s bat	15	Lesion	05/04/19	Jersey Zoo	1	JAELZA000000000

27175_54M3289Y3	Livingstone’s bat	15	Lesion	05/04/19	Jersey Zoo	1	JAELYZ000000000
27176_54M3289Y4	Livingstone’s bat	15	Lesion	05/04/19	Jersey Zoo	1	JAELYY000000000
27177_54M3289Y5	Livingstone’s bat	15	Lesion	05/04/19	Jersey Zoo	1	JAELYX000000000
27185_155F122	Livingstone’s bat		Faeces	15/05/19	Comoros	1	JAELYW000000000
27196_175F314	Livingstone’s bat		Faeces	17/05/19	Comoros	1	JAELYV000000000
27194_175F303	Livingstone’s bat		Faeces	17/05/19	Comoros	1	JAELYU000000000
27195_175F313	Livingstone’s bat		Faeces	17/05/19	Comoros	1	JAELYT000000000
27197_175F322	Livingstone’s bat		Faeces	17/05/19	Comoros	1	JAELYS000000000
27200_175F351	Livingstone’s bat		Faeces	17/05/19	Comoros	1	JAELYR000000000
27201_175F382	Livingstone’s bat		Faeces	17/05/19	Comoros	1	JAELYQ000000000
27192_155F261	Livingstone’s bat		Faeces	15/05/19	Comoros	1	JAELYP000000000
27198_155F12	Livingstone’s bat		Faeces	15/05/19	Comoros	1	JAELYO000000000
27204_175F463	Livingstone’s bat		Faeces	17/05/19	Comoros	1	JAELYN000000000
27206_175TM551	Livingstone’s bat		Chewed fruit	17/05/19	Comoros	1	JAELYM000000000
27193_175F292	Livingstone’s bat		Faeces	17/05/19	Comoros	1	JAELYL000000000
27205_175F522	Livingstone’s bat		Faeces	17/05/19	Comoros	1	JAELYK000000000
27199_155F114	Livingstone’s bat		Faeces	15/05/19	Comoros	1	JAELYJ000000000
27191_155F222	Livingstone’s bat		Faeces	15/05/19	Comoros	1	JAELYI000000000
27188_155F133	Livingstone’s bat		Faeces	15/05/19	Comoros	1	See closed genome
27186_155F131	Livingstone’s bat		Faeces	15/05/19	Comoros	1	JAELYH000000000
27184_155F92	Livingstone’s bat		Faeces	15/05/19	Comoros	1	JAELYG000000000
27181_155F72	Livingstone’s bat		Faeces	15/05/19	Comoros	1	JAELYF000000000
30406_155F136	Livingstone’s bat		Faeces	15/05/19	Comoros	1	JAELYE000000000
30412_155F202	Livingstone’s bat		Faeces	15/05/19	Comoros	1	JAELYD000000000
NW LYH	Livingstone’s bat	1	Skin	14/07/15	Jersey Zoo	1460	JAELYB000000000
NC LYH	Livingstone’s bat	1	Lesion	14/07/15	Jersey Zoo	1460	JAELYC000000000
nan_175_F371_ch*	Livingstone’s bat		Faeces	17/05/19	Comoros	3926	CP066492-CP066495
ncr_155_F133*	Livingstone’s bat		Faeces	15/05/19	Comoros	1	CP066488-CP066491

*Closed genome

Supplementary Table S2; Plasmids detected in each isolate using Mob-Suite. Number of plasmids, suggested replicon type and accession number of replicon.

Strain ID	Sequence Type	Number of plasmids	Replicon Types	Accession numbers
18/1-F BHC	3926	1	rep_cluster_1017	000261__GQ900456
27178_155F43	3926	1	798	NC_025175
27179_155F52	3926	3	798, rep_cluster_1257, 1733	NC_025175, 000569__NC_020165_00005, 001150__NC_013330_00017
27182_155F75	3926	1	rep_cluster_1733	001150__NC_013330_00017
27187_155F132	3926	1	rep_cluster_1733	001150__NC_013330_00017
27189_155F174	3926	3	rep_cluster_798, 1257, 1733	NC_025175, 000569__NC_020165_00005, 001150__NC_013330_00017
27190_155F201	3926	1	rep_cluster_1017	000261__GQ900456
27202_175F371	3926	3	rep_cluster_1017, 1230, 1017	000259__NC_013375_00001, 000529__NC_007768_00001, 000261__GQ900456
27203_175F433	3926	3	rep_cluster_1017, 1230, 1017	000259__NC_013375_00001, 000529__NC_007768_00001, 000261__GQ900456
27207_175TM671	3926	3	rep_cluster_1017, 1230, 1017	000259__NC_013375_00001, 000529__NC_007768_00001, 000261__GQ900456
30404_155F173	3926	2	rep_cluster_1733, 1257	001150__NC_013330_00017, 000569__NC_020165_00005
30407_155F163	3926	3	rep_cluster_1230, 1733, 1257	000529__NC_007768_00001, 001150__NC_013330_00017, 000569__NC_020165_00005
30408_155F165	3926	2	rep_cluster_1733, 1257	001150__NC_013330_00017, 000569__NC_020165_00005
30409_155F184	3926	2	rep_cluster_1733, 1257	001150__NC_013330_00017, 000569__NC_020165_00005
30411_155F73	3926	1	rep_cluster_1733	001150__NC_013330_00017
27/4-18 LC	1	1	rep_cluster_469	NA
27/4-20 AHC	1	1	rep_cluster_936	002421__GU237136_00023
27181_155F72	1	1	rep_cluster_1017	000259__NC_013375_00001
27184_155F92	1	2	rep_cluster_1017, 1017	000259__NC_013375_00001, 000261__GQ900456
27185_155F122	1	2	rep_cluster_1017, 1017	000259__NC_013375_00001, 000261__GQ900456
27186_155F131	1	2	rep_cluster_1230, 1017	000529__NC_007768_00001, 000261__GQ900456
27188_155F133	1	2	rep_cluster_1230, 1017	000529__NC_007768_00001, 000261__GQ900456
27191_155F222	1	1	rep_cluster_1017	000261__GQ900456
27192_155F261	1	1	rep_cluster_1017	000259__NC_013375_00001
27193_175F292	1	1	rep_cluster_1017	000261__GQ900456
27194_175F303	1	2	rep_cluster_1017, 1017	000259__NC_013375_00001, 000261__GQ900456
27195_175F313	1	2	rep_cluster_1017, 1017	000259__NC_013375_00001, 000261__GQ900456
27196_175F314	1	2	rep_cluster_1017, 1017	000259__NC_013375_00001, 000261__GQ900456
27197_175F322	1	2	rep_cluster_1017, 1017	000259__NC_013375_00001, 000261__GQ900456
27198_155F12	1	1	rep_cluster_1017	000259__NC_013375_00001
27199_155F114	1	1	rep_cluster_1017	000261__GQ900456
27200_175F351	1	2	rep_cluster_1017, 1017	000259__NC_013375_00001, 000261__GQ900456
27201_175F382	1	1	rep_cluster_1017	000259__NC_013375_00001
27204_175F463	1	2	rep_cluster_155, 1017	000915__CP001784_00003, 000261__GQ900456
27205_175F522	1	2	rep_cluster_1230, 1017	000529__NC_007768_00001, 000261__GQ900456
27206_175TM551	1	2	rep_cluster_155, 1017	000915__CP001784_00003, 000261__GQ900456
30406_155F136	1	2	rep_cluster_1230, 1017	000529__NC_007768_00001, 000261__GQ900456
30412_155F202	1	1	rep_cluster_1017	000261__GQ900456
ncr_155_F133 *	1	2	rep_cluster_1230, 1017	000529__NC_007768_00001, 000529__NC_007768_00001
nan_175_F371_ch *	3926	3	rep_cluster_1017, 1017, 1230	000261__GQ900456, 000259__NC_013375_00001, 000529__NC_007768_00001

Plasmids predicted by Mob-suite in each strain. * indicates completed genomes.
